# Taxa Atual de Mortalidade por Doenças Cardiovasculares no Estado do Rio de Janeiro: Mais do que Apenas um Sonho no Rio

**DOI:** 10.36660/abc.20210203

**Published:** 2021-04-08

**Affiliations:** 1 Universidade de São Paulo Faculdade de Medicina Instituto do Coração São PauloSP Brasil Instituto do Coração (InCor), Faculdade de Medicina, Universidade de São Paulo, São Paulo, SP - Brasil.

**Keywords:** Doenças Cardiovasculares/mortalidade, Isquemia Miocárdica, Fatores de Risco, Hipertensão, Obesidade, Dislipidemia, Diabetes Mellitus, Fatores Socioeconômicos, Educação

As doenças cardiovasculares (DCV) são as principais causas de morte em mulheres e homens no Brasil. Desde 1980, houve uma redução significativa da mortalidade por essas doenças. De 1980 a 2012, a menor redução foi de 31% para doenças isquêmicas do coração (DIC) em homens, e a maior redução foi de 54% para doenças cerebrovasculares (DCBV) em mulheres.^1^ Apesar de uma redução importante da mortalidade por DIC, a redução da mortalidade por DCBV foi a que mais contribuiu para a redução total da mortalidade por DCV. No entanto, análises comparativas dos períodos de 1980 a 2006 com os de 2007 a 2012 mostraram maior redução percentual da mortalidade por DCV, DIC e DCBV no período de 1980 a 2006. No período de 2007 a 2012, houve redução significativa, porém menos intensa, da mortalidade por DCV e DCBV quando comparada ao período anterior, enquanto a mortalidade por DIC permaneceu inalterada em mulheres e homens. O mesmo fenômeno foi observado nos EUA e em alguns países da Europa neste período, e essa tendência desfavorável tem sido associada ao aumento da incidência de obesidade e diabetes mellitus e ao controle inadequado dos fatores de risco.^2^^–^^4^ O controle dos principais fatores de risco para DCV reduz a mortalidade por DCV em pelo menos 50%, e o que se destaca neste processo é a prevenção primária por meio do controle dos principais fatores de risco, a saber, hipertensão, tabagismo, diabetes e dislipidemia.^5^^,^^6^

A hipertensão é o principal fator de risco na gênese da DCBV, enquanto os demais fatores de risco também participam da gênese da DIC, o que leva a maior dificuldade na prevenção da DIC, justificando a tendência desfavorável da DIC em relação à DCBV. Atualmente, o nível socioeconômico (renda familiar, emprego, educação e fatores ambientais) também é considerado um fator de risco independente para DCV que é equivalente aos fatores de risco tradicionais.^7^ A influência do nível socioeconômico é um dos principais fatores responsáveis pela maior mortalidade por DCV nas populações menos favorecidas.

Um estudo recente mostrou uma redução acentuada da mortalidade por DIC e DCBV nas regiões mais desenvolvidas do Brasil (Sudeste e Sul), o que não foi observado nas demais regiões do país.^8^ Apesar disso, mesmo as regiões Sudeste e Sul apresentam microrregiões bastante heterogêneas do ponto de vista socioeconômico e dados bastante heterogêneos de mortalidade por DCV. Um estudo de Rosa et al. mostrou essa heterogeneidade na mortalidade por DCV em regiões de saúde do estado do Rio de Janeiro, incluindo a capital.^9^ As regiões de saúde foram definidas como “espaço geográfico contínuo constituído por grupos de municípios limítrofes, delimitados com base em identidades culturais; redes econômicas, sociais e de comunicação; e infraestrutura de transporte compartilhada”. Relataram, em pelo menos 50% das regiões, tendências desfavoráveis na mortalidade prematura (30 a 69 anos) e tardia (≥ 70 anos) por DCV em períodos específicos de 1996 a 2016. Em geral, os coeficientes de mortalidade ajustados para DCV, DIC e DCBV tiveram redução significativa, para todas as regiões, em mulheres e em homens, quando foram usados zero pontos de junção no Programa de Regressão Joinpoint^10^ (Tabela 1). Porém, quando utilizaram um ou mais pontos de junção, ou seja, dividiram a reta de regressão total que correspondia a todo o período de 1996 a 2016 em dois ou mais períodos, encontraram períodos específicos onde mortalidade prematura e tardia por DIC e DCBV aumentaram ou permaneceram estáveis. Quase todos esses períodos específicos foram dos últimos anos analisados, do período de 1996 a 2016. A análise desses períodos específicos mostrou que praticamente todas as regiões de saúde tiveram resultados desfavoráveis na mortalidade por DCV, DIC e DCBV, com as seguintes exceções: mortalidade prematura por DCV em mulheres na cidade de Rio de Janeiro, todas as mortes por DCV em homens na Baixada Litorânea, DIC em mulheres na cidade de Rio de Janeiro e na Baixada Litorânea e mortalidade tardia por DIC nas regiões Metropolitana 2 e Noroeste. Nas demais regiões, de acordo com os dados disponíveis na Tabela 2, foram observadas tendências desfavoráveis na mortalidade por DCV em praticamente todo o estado de Rio de Janeiro. Os autores não avaliaram as causas destes resultados desfavoráveis; sugeriram a influência de aspectos socioeconômicos e o controle inadequado dos fatores de risco, o que provavelmente deve ter ocorrido. No entanto, faltaram alguns dados sobre a mortalidade por DCV em algumas regiões de saúde, particularmente em relação à DCBV em mulheres.

Em suma, as melhorias das condições socioeconômicas e a intensificação dos programas de prevenção primária para as DCV são essenciais para reverter essas tendências tardias da mortalidade por DCV no estado do Rio de Janeiro.
