# Supplementary material for: Taxa Atual de Mortalidade por Doenças Cardiovasculares no Estado do Rio de Janeiro: Mais do que Apenas um Sonho no Rio
Source: Arq Bras Cardiol. 2021 Apr 8;116(4):772–3. [Article in Portuguese] doi: 10.36660/abc.20210203 (PMC8121397; doi:10.36660/abc.20210203)
Supplement: Translation

# Translation: Current Cardiovascular Disease Death Rate in Rio De Janeiro State: More than Only a Dream in Rio

**Affiliations:** 1 São PauloSP Brazil Instituto do Coração (InCor), Faculdade de Medicina, Universidade de São Paulo, São Paulo, SP - Brazil.

**Keywords:** Cardiovascular Diseases/mortality, Myocardial Ischemia, Risk Factors, Hypertension, Dyslipidemia, Diabetes Mellitus, Socioeconomics Factors, Education

Cardiovascular diseases (CVD) are the main causes of death in women and men in Brazil. Since 1980, there has been a significant reduction in mortality from these diseases. From 1980 to 2012, the smallest reduction was 31% for ischemic heart diseases (IHD) in men, and the largest reduction was 54% for cerebrovascular diseases (CBVD) in women.^1^ Despite an important reduction in mortality due to IHD, the reduction in mortality due to CBVD was the one that most contributed to the total reduction in mortality due to CVD. However, comparative analyses of the periods from 1980 to 2006 with those from 2007 to 2012 showed a greater percentage reduction in mortality from CVD, IHD, and CBVD in the period from 1980 to 2006. In the period from 2007 to 2012, there was a significant, but less intense, reduction in mortality from CVD and CBVD when compared to the previous period, while mortality from IHD remained unchanged in women and men. The same phenomenon was observed in the USA and in some European countries for this period, and this unfavorable trend has been associated with the increase in the incidence of obesity and diabetes mellitus and with the inadequate control of risk factors.^2^^–^^4^ The control of the main risk factors for CVD reduces CVD mortality by at least 50%, and the highlight in this process is primary prevention by controlling the main risk factors, namely hypertension, smoking, diabetes, and dyslipidemia.^5^^,^^6^

Hypertension is the main risk factor in the genesis of CBVD, while the other risk factors also participate in the genesis of IHD, which leads to greater difficulty in preventing IHD, justifying the unfavorable tendency of IHD compared to CBVD. Currently, socioeconomic status (family income, employment, education, and environmental factors) is also considered an independent risk factor for CVD that is equivalent to traditional risk factors.^7^ The influence of socioeconomic status is one of the main factors responsible for the highest mortality from CVD in the least favored populations.

A recent study showed a marked reduction in mortality from IHD and CBVD in the most developed regions of Brazil (Southeast and South), which was not observed in other regions of the country.^8^ Nevertheless, even the Southeast and South regions have very heterogeneous microregions from the socioeconomic point of view and rather heterogeneous death rate data from CVD. A study by Rosa et al. showed this heterogeneity in CVD mortality in health regions of the state of Rio de Janeiro, including the capital city.^9^ Health regions were defined as “continuous geographic space consisting of groups of bordering municipalities, delimited based on cultural identities, economic and social and communication networks and shared transport infrastructure.” They reported, in at least 50% of regions, unfavorable trends in premature (30 to 69 years) and late (≥ 70 years) mortality from CVD in specific periods from 1996 to 2016. In general, the adjusted mortality coefficients for CVD, IHD, and CBVD had a significant reduction for all regions in women and in men, when zero junction points were used in the Joinpoint Regression Program^10^ (Table 1). However, when the authors used one or more junction points, that is, they divided the total regression line corresponding to the entire period from 1996 to 2016 in two or more periods, they found specific periods where premature and late mortality from IHD and CBVD increased or remained stable. Almost all of these specific periods were from the last years analyzed, from the period from 1996 to 2016. The analysis of these specific periods showed that practically all health regions had unfavorable results in mortality from CVD, IHD, and CBVD, with the following exceptions: premature CVD mortality in women in the city of Rio de Janeiro, all CVD death in men in the Baixada Litorânea region, IHD in women in the city of Rio de Janeiro and the Baixada Litorânea region, and late mortality due to IHD in the Metropolitan 2 and Northwest regions. In the other regions, according to the data available in Table 2, unfavorable trends in CVD mortality were observed in practically the entire state of Rio de Janeiro. The authors did not evaluate the causes of these unfavorable results; they suggested the influence of socioeconomic aspects and the inadequate control of risk factors, which probably must have occurred. Nevertheless, some data on CVD mortality were missing for some health regions, particularly in relation to CBVD in women.

In summary, improvements in socioeconomic conditions and the intensification of primary prevention programs for CVD are essential to reverse these late trends of CVD mortality in the state of Rio de Janeiro.
